# Reflecting on Themes From Telehealth Consultations Early in the Pandemic: An Opportunity to Learn From Multiprofessional Colleagues

**DOI:** 10.1155/ijta/7172059

**Published:** 2026-04-29

**Authors:** Maggie Meeks, Laura R. Joyce, John Dean, Lorna Davies, Tika Ormond, Philippa Seaton, Dale Sheehan, Dean Sutherland, Melanie Welfare, Arindam Basu

**Affiliations:** ^1^ Office of the Chief Medical Officer, Health New Zealand, Christchurch, New Zealand; ^2^ Department of Surgery and Critical Care, University of Otago Christchurch, Christchurch, New Zealand, otago.ac.nz; ^3^ Department of Primary Care and Clinical Simulation, University of Otago Christchurch, Christchurch, New Zealand, otago.ac.nz; ^4^ School of Midwifery, Otago Polytechnic, Dunedin, New Zealand; ^5^ School of Psychology, Speech and Hearing, University of Canterbury, Christchurch, New Zealand, canterbury.ac.nz; ^6^ Department of Nursing, University of Otago Christchurch, Christchurch, New Zealand, otago.ac.nz; ^7^ Medical Education Unit, University of Otago Christchurch, Christchurch, New Zealand, otago.ac.nz; ^8^ School of Nursing and Midwifery, Edith Cowan University, Perth, Australia, ecu.edu.au; ^9^ School of Health Sciences, University of Canterbury, Christchurch, New Zealand, canterbury.ac.nz

## Abstract

**Aims:**

This study is aimed at documenting the reflections of New Zealand healthcare professionals on the use of synchronous telehealth consultations.

**Methods:**

A qualitative narrative inquiry was conducted to explore the practice of telehealth in New Zealand. Purposive sampling was used to identify clinicians from multiple professions who used telehealth during the initial phase of the COVID‐19 pandemic. Fifteen semistructured interviews were conducted between October 2020 and May 2021 with clinicians from primary and secondary care, including multiple professional backgrounds. Interview transcripts were analysed thematically.

**Results:**

Six themes were identified: (1) equitable access: There were concerns regarding equitable access to telehealth; (2) relationships and connections: This included connection with the wh*ā*nau/family and their culture, between professions and as part of the wider health system; (3) information gathering and sharing: This included the visibility of the process as well as visibility of information regarding the client/patient; (4) adapting to change: There was significant variation between clinicians in transitioning to using telehealth; (5) professional boundaries: This included the prescribed boundaries such as the physical location of patients/clients, as well as unanticipated changes to personal, professional and organisational boundaries; (6) IT logistics: This included the potential technological drivers (enablers and disablers) within the process of incorporating telehealth.

**Conclusions:**

Telehealth was critical in healthcare provision during the COVID‐19 pandemic and has continued to be used within healthcare delivery postpandemic. The themes identified provided insight into the importance of considering the provision of telehealth as a complex package and identifying contextual challenges as well as the enablers and potential benefits of using this modality.

## 1. Introduction

Using telehealth in healthcare provision was an important component of the international health response during the COVID‐19 pandemic. The use of telehealth differed in various settings and populations; it has been reported that 9% of general practice (GP) visits converted from face‐to‐face to telemedicine in the early pandemic in Australia [1]. In the United States, telehealth use quadrupled for the care of older adults [2], and in rural Canada, there was a tenfold increase in the use of telehealth [3]. In New Zealand, there have been proponents for telehealth since the end of the last century [4]. This had enabled rural hospitals to connect with secondary and tertiary urban hospitals for specialist input, but uptake of such video‐conferencing solutions in routine practice was exceptional rather than the norm [5] [6]. The World Health Organization (WHO) had also produced a comprehensive guide for the implementation of telemedicine in 2016, and New Zealand published its own guide for establishing and maintaining sustainable telemedicine services in 2017 [7] [8]. Despite these resources, there were relatively few trained professionals experienced in the delivery of this type of care at the beginning of 2020 [9].

The terms telehealth and telemedicine are often used interchangeably, but an accepted definition of telemedicine is ‘the remote diagnosis and treatment of patients by means of telecommunications technology’, while the definition of telehealth has been expanded to encapsulate a broader array of digital healthcare activities and services [10, 11]. Telehealth is the term that will be used in this paper, and in this study, we are referring specifically to the use of virtual synchronous consultations. Primary indications for telehealth are to deliver remote care where the clients and the providers are separated over distance (where the challenges include geography/transport networks/expeditions/natural disasters/civil conflict/time). In these contexts, telehealth facilitates the ongoing delivery of care either synchronously (virtual consult) or asynchronously, where digitised images and data would be accessed at a different time (known as store and forward).

On 30/01/2020, the WHO declared COVID‐19 a public health emergency of international concern (PHEIC) [12]. In order to contain community transmission of SARS‐COV‐2 virus and the resultant COVID‐19 infection, and as part of an elimination plan, several countries enforced social isolation and distancing and used a ‘lockdown’ strategy (where movement between areas was restricted) [13]. As a result, face‐to‐face consultations for medical care had to be minimised and in many instances were replaced with virtual consultations. In New Zealand, the provision of emergency healthcare needed to coexist with public health measures that included self‐isolation and formation of isolated community groups (known as ‘bubbles’), and there were challenges in providing routine care and preventive health services. This resulted in a rapid uptake of telehealth practices in the setting of GP, community and hospital care with limited preparation time. The New Zealand Government adopted a four‐level tiered alert system to specify public health and social measures to address the COVID‐19 pandemic [14]; during the highest levels of the COVID‐19 lockdown, many healthcare professionals across all spectrums of the health service in New Zealand found themselves using online video/audio calling services. This included transitioning from ‘in‐person’ consultations to the use of telehealth with clients/patients. It is this use of telehealth that is the focus of the work reported here, in the context of synchronous consultations. This paper contributes knowledge from a study that was conducted across professional, departmental and organisational boundaries.

Our primary aim was to document the experiences and reflections of colleagues from different professions and areas of healthcare in the use of synchronous telehealth. This research particularly explored the views of New Zealand–based health practitioners from diverse clinical backgrounds regarding their professional experiences of telehealth during Levels 3 and 4 of the nationwide COVID‐19 lockdown in 2020. Beyond the immediate context of the pandemic, these findings may serve as a critical reflection on telehealth integration that can be leveraged to safeguard continuity of care and improve system agility in anticipation of future large‐scale healthcare disruptions.

## 2. Methods

### 2.1. Design

The Consolidated Criteria for REporting Qualitative studies (COREQ) was used in this study (see Appendix 1). This project was a qualitative narrative inquiry into practice using semistructured interviews with health practitioners about their recent telehealth experiences. At each stage of the project, there was collaboration of the interprofessional group of researchers (the ‘study team’). The majority of the study team also performed interviews of study participants. The study team collaborated in the design of the semistructured questions, discussion of personal perspectives on themes and the validation of the final thematic framework.

### 2.2. Background

Te Papa Hauora (https://www.healthprecinct.org.nz/) was established in Christchurch, New Zealand, in 2014. In 2019, an Educational Research Forum was developed within Te Papa Hauora with the aim of bringing together those involved in both undergraduate and postgraduate education across professions and organisations to develop a collaborative forum for research innovation and sustainability. As a group, we sought to move beyond networking to building a dual professional and interprofessional identity. This dual identity refers to the simultaneous development of a robust sense of belonging to both one’s own profession and to the interprofessional community of healthcare [15] [16]. This strategy is aimed at building a collaborative understanding of each other’s professional, educational and research perspectives ‘with, from and about’ each other in the process of doing research [17].

The two study team leads (DS/MGM) invited colleagues from the Health Precinct Educational Research Forum as well as other colleagues interested in the area of telehealth to develop a research project which is aimed at investigating the experiences of clinicians in synchronous telehealth consultations during the New Zealand period of lockdown. An interprofessional methodology was developed with the intention of developing interprofessional understanding within the research group and the capability of each professional researcher in the area of qualitative interprofessional research.

### 2.3. Development of Semistructured Interview Questions

All members of the interprofessional research team contributed to the development of the semistructured questions by participating in face‐to‐face brainstorming sessions facilitated by a researcher experienced in this methodology (DS). This session included a robust discussion on the interprofessional perspectives of language and terminology (e.g., patient vs. client) and alternative views of practice (e.g., discussion of the viewpoints of participating professionals regarding terms such as health) to ensure that the questions were worded to be professionally inclusive. The questions were refined and then trialled by two of the study team (DS and DSu) before being sent back to the group until a consensus was reached. The leaders of the creation of the interview guide have extensive experience in both medical education and interprofessional education for over 30 years, and DS has a background in radiography, and DSu has a background in speech and language therapy (SLT).

### 2.4. Conducting Interviews

The study team interviewers were encouraged to find one to three interviewees either from their own professional/clinical field or a work area that they were familiar with (e.g., midwives and lactation consultants; colleagues from different professional backgrounds working together in primary care, such as speech language therapists and general practitioners). This often meant that they interviewed a colleague in their own zone of practice, but not necessarily from the same profession. This allowed the interviewer to combine an element of naive enquiry with an enhanced understanding because of their knowledge of the practice environment. There were no specific exclusions to participation. While no formal a priori sample size was calculated, recruitment followed a pragmatic, purposive approach to ensure a range of professional perspectives. Data collection continued until the research team observed significant thematic redundancy, at which point further interviews were unlikely to yield substantially new insights.

The interviews were conducted using a variety of modalities that included in‐person, by phone (with and without video images), or over Zoom and were all recorded (either using Zoom or separate audio) with the participant’s written consent. The mode of interviews was what best suited both interviewee and interviewer by mutual agreement. This was a practical approach for a COVID‐19 environment and at the time was a familiar and comfortable environment for all and mirrored the research agenda. The video and audio recordings were downloaded and stored in a secure online environment with password access before being transcribed by one researcher (DS). The transcripts were stored in the same secure online environment and were made available for the thematic analysis by the two study team leads (DS/MGM). Transcripts were returned to participants for checking for those who chose to do this.

### 2.5. Theme Development

The two study team leads, one with a radiography background and one with a medical background, followed a process of independent data familiarisation, by the in‐depth review of transcripts, to derive preliminary themes in an inductive interpretative approach [18] [19]. This research strategy involved the multidisciplinary research team moving from specific data observations toward broader thematic generalisations, seeking to understand the subjective meanings and social realities constructed by participants within a specific context. These two faculty members then met to review and label their initial themes before continuing a process of independent review followed by collaborative review until they were satisfied that there were five *potential* themes (inequity, connection, unspoken information, novice to expert, and boundaries and innovation).

These five initial themes were taken to the wider interviewer group for further discussion. The interdisciplinary interview team met in person and virtually to talk through the potential themes with reference to their own experiences in interviewing. The themes were refined and expanded to six during this process. The final stage involved member checking, where all study team members reviewed two or three transcripts (excluding the transcript(s) of the interviews they performed) and the proposed themes.

In developing these themes, we went beyond having a multiprofessional research team to developing a research team that continually improved their understanding of the professional identities, roles and language of other professions as both practitioners and researchers. We challenged assumptions and professional biases that appeared to arise from the individual’s own profession’s lens. In this methodology, we are valuing and prioritising that we really understand and respect others’ professional perspectives while learning about their practice.

### 2.6. Ethics

Ethical approval (HEC 2020/52) was received in September 2020 from the University of Canterbury Human Ethics Committee, and updates to this were obtained to allow an interviewee from outside of Canterbury to be interviewed. An information sheet and consent form were provided for those being interviewed, who were made aware that the only personal information collected was profession and gender. This research was educational research conducted with healthcare practitioners.

## 3. Results

### 3.1. Demographics

There were 10 study team members, of whom 9 conducted interviews between October 2020 and May 2021. A total of 15 interviews were recorded and transcribed for analysis. Interviews lasted between approximately 30 and 90 min. The study team members worked in rural primary care, medicine, midwifery, nursing, medical radiation technology, and speech language therapy. The study team members included both males and females and had 15 or more years of clinical experience. In addition, a number of the team members have postgraduate education qualifications.

The study participants were from a variety of health professions working in primary and secondary care, GP/developmental paediatrics (DevP) and general paediatrics (GenP)/community neonatal nurse (CNN)/child psychiatrist (CP)/midwifery (with roles as lead maternity carers, core midwives and lactation consultant [MW])/neurologist (Neuro)/speech and language therapy (SLT) and included Māori representation (GP and LC).

### 3.2. Themes

There were six main overarching themes identified as follows:

• Equitable access
• Relationships and connections
• Information gathering and sharing
• Adapting to change
• Professional boundaries
• Information technology (IT) logistics

These themes are described below with their subthemes and examples of quotes from the transcribed interviews.

#### 3.2.1. Theme 1: Equitable Access

Access to telehealth was not perceived by the healthcare practitioners interviewed as part of this study to be equitable for the client/patient. The causes of the inequity ranged from factors within the client’s own specific context (e.g., school‐aged children requiring access at the same time and limited devices or limitations of home network) through to the more general availability of internet access and organisation of healthcare systems (e.g., aged residential or nursing level care) and processes; a common concern was that access was limited by individual finances that included the capital outlay of purchasing devices as well as the operational costs (‘everyone does not have access to the internet’ [MW], ‘if you have no money on your phone, you can’t text back’ [MW]). The influence of geography on internet access was also raised (‘some areas are not internet friendly…patients had to come into the rural hospital’ [SLT]). The issue of cultural equity was raised; there were government instructions to limit time spent in face‐to‐face sessions which did not align with the cultural expectations of some of our clients (e.g., indigenous Māori as well as some immigrant populations) (‘culturally it is really rude to rush in’ [MW]). There were concerns, particularly from those working in primary care, that certain individuals—including those already disadvantaged by existing barriers to healthcare—were more at risk of poor health outcomes because of the prioritisation of telehealth over face‐to‐face consultations (‘our most vulnerable patients cannot get access…get left behind again’ [GP]). Some participants perceived improved access to healthcare and healthcare resources because of telehealth, but several participants noted an exaggeration of pre‐existing inequity.

#### 3.2.2. Theme 2: Relationships and Connections

In this theme, there was exploration of the influence of telehealth on relationships or the concept of connecting with others; this included the personal connection within the professional context, connecting with the wider wh*ā*nau/family, and provision of cultural safety or connecting as part of the wider health system. The telehealth consultation seemed to be more acceptable where the patient/client was already known to the professional especially for follow‐up discussions and sharing the results of investigations (‘we tended to focus on follow up…just to make sure that they were stable and ok’ [Neuro], ‘If I have met the person prior to the sessions that might work well’ [SLT]). In consultations with children, there was some evidence that a virtual consultation could enhance the development of connection (‘some older kids talked more than they would have in person’ [GP]). The virtual consultation could yield enhanced connectivity because of a shared experiential context (‘because I am not sitting in someone else’s home…I’m sitting in mine and they are in theirs and we have that connection’ [SLT]). The situation could be more complex with clients/patients that had particular challenges (‘face‐to‐face is much better especially with mental illness’ [GP], ‘a barrier with people that struggle with relationships’ [GP]) or where a relationship had not already been established (‘Midwifery is too much about the relationship with people…does not feel right through the computer screen’ [MW]). This was also the case when the process of the consultation with a client/patient and the possible outcome of the discussion was likely to be upsetting, or sensitive medical information, diagnoses or prognoses were shared. There were also some situations where a particular connection had not previously been available or where barriers to connecting had been removed; this included involving other family members who lived separately in a discussion or involving other professionals that worked in different organisations.

#### 3.2.3. Theme 3: Information Gathering and Sharing

This theme included aspects such as the visibility of the process as well as the visibility of information regarding the client or patient. If an examination or immediate treatment were felt to be a possibility, then telehealth was not suitable (‘the examination itself was the clincher some times’ [Neuro], ‘one woman said breastfeeding was going fine…but when I got there [it wasn’t going fine]…I gave up on Zoom for postnatal’ [MW]). In contrast, the digital space could provide more information in some specific situations such as when a child’s behaviour or development could be directly observed within a physical space that felt comfortable to them (‘for a lot of kids it worked well, especially ADHD follow up…actually that they are in their own home’ [GP], ‘Child comfortable at home no stress….video on child able to work through diagnostic questions’ [Dev Paed]). Some clinicians talked of making clear changes in their own consultation patterns (‘on the phone have to follow a structure’ [GP]) and others recognised the opportunity to partner with clients by sharing their screen and identifying educational resources that they could access during consultations to facilitate their discussions with the client (‘using screenshots from the BreastfedNZ app and Mama Aroha talk cards were very helpful’ [MW]). The importance of continuing to provide cultural safety was also articulated (‘came up with a tele hui process’ [GP]). There was some concern about the fact that nonverbal aspects of communication were not as visible (‘lots of missed opportunities when things are done by phone or online’ [MW]). A significant concern for those clinicians who reviewed at home was also the psychosocial context (including privacy) to which the client/patient was exposed (‘can be in violent homes or drug use…seeing them is important…comes down to social issues’ [MW], ‘you have no idea if anyone else is listening’ [MW]). Some clinicians took specific steps to try to protect the confidentiality of their clients/patients (‘my family on strict instructions not to come into the bedroom until door open ‐ briefed them about not listening etc.’ [MW]).

#### 3.2.4. Theme 4: Adapting to Change

There was significant variation between clinicians as they incorporated telehealth into their practice, which may have been influenced by their own attitudes and belief systems, as well as external organisational factors [20]. Clinical decision‐making was influenced by factors such as their perceived priorities in patient/client care (‘I was one who kept seeing my women…you couldn’t just leave them’ [MW]) and their clinical or technological experiences (‘I put out a message asking if anyone was interested in doing a free online support’ [MW]). Other innovations included a hospital staff member creating a professional Facebook account to connect with new mothers that enabled them to share photos and videos and a speech and language therapist using a decibel reader as an *audiotool* to help evaluate a client’s speech volume. In some situations, the timing of the consultation was more convenient (‘with normal school times, I would have had to have done at either side of that time’ [SLT]). Some comments showed how the use of telehealth could be integrated efficiently into the wider context of their professional role (‘most often did them in my car while driving somewhere’ [MW], ‘so a lot of scaffolding up with very short verbal appointments’ [LMC]). Some of this practice has been continued (‘after lockdown the client we did by Zoom when she did not want to travel’ [MW]). In some situations where a clinical examination and specialist opinion were necessary, the telehealth consultation could be less efficient (‘had to have another doctor (on site) examining the patient and so two doctors were taken out’ [Neuro]). While some healthcare employers were felt to be less adaptable, it was encouraging to hear of the adaptability of government organisations such as Work and Income New Zealand (WINZ, that provides financial support to beneficiaries in New Zealand) and the Accident Compensation Corporation (ACC) (a scheme which supports the healthcare and ongoing costs for those who sustain an injury) (‘work and income…kept all benefits going’ [GP], ACC and Work and Income were very supportive [GP]).

#### 3.2.5. Theme 5: Professional Boundaries

This theme includes the unanticipated changes to personal, professional and organisational boundaries. It is not uncommon when visiting the General Practitioner in New Zealand to bring multiple complaints to the same appointment. It was noted that this did not occur when people used telehealth as they tended to prioritise their most important need. (‘people did not come with lots of extra complaints – they got right to the essence of the problem’ [GP]). The patients/clients did not always respond to a cold call despite the likelihood of being at home as a result of the government‐imposed COVID‐19 restrictions (‘one day I did sort of cold calls…out of 10 I got 2’ [GP]). This may have been influenced by several factors, including access (as mentioned in *Theme 1*), and was also true for the clinicians themselves (‘my partner was at home, he had Zoom meetings for work, kids for school…a juggle how many were on the internet at times’ [MW]). This extension of work into home life also had financial consequences (‘I got caught out when their audio died…(my) phone bill was $200 that month’ [MW]) and the importance in putting in boundaries was described (‘it is about respecting that phone as a business tool and not a 24 hr attachment’ [MW], ‘they (messages) were coming in all hours – high anxiety for myself’ [MW]). Clinical processes also became blurred (‘cut corners…did not worry about red tape’ [MW], ‘we did some prescribing that had been prescribed before by the GP but usually would not have’ [MW]).

#### 3.2.6. Theme 6: IT Logistics

This final theme focused on the complexity of the telehealth processes. Those interviewed talked about the lack of an *effective* infrastructure for healthcare staff (e.g., useful guidelines, software availability and IT support) as well as appropriate education possibilities and opportunities to practice. Many of the clinicians talked of guidelines that came from a variety of sources which may have contradicted each other (‘it took a while for any guidance to come through…at times (these) contradicted each other’ [MW], ‘college more practical for the Midwife…the MOH not realistic…not understand how Midwives work’ [MW]). Social media and peer support often appeared to be more helpful (‘looked to social media and what others were doing’ [MW], ‘having group support was beneficial when life was changing fast’ [MW]). Training was mentioned (‘need ongoing education and utilisation of technologies’ [Neuro], ‘it wasn’t, you know, a formal type of training…it was how do I do this thing and show me’ [SLT]). The organisation of the virtual consultation came either ‘reactively’ at the clients/patient’s request or ‘proactively’ at the clinician’s request, and the clinician usually had significant input into the organisation of the consultation although some that worked in larger organisations had administrative support (‘be organised before call…have a backup option’ [MW], ‘sometimes easier to send a link right at that moment ‐ an appointment too far in advance did not work’ [CNN]). The most common choice of technology was the telephone, and some have continued this (‘some people have continued this now…say can I please have a phone consultation’ [GP]). There were a variety of opinions about using video links (‘face felt so close…self‐conscious…I stopped as not concentrating’ [MW]), ‘recognise situations where it does not work’ [Neuro]) and the importance of catering to all (those that ‘do not fit in the box…Māori / Pacific / low socio‐economic or those living off the grid’ [MW]).

## 4. Discussion

This study reports healthcare professionals’ experiences of telehealth during the initial phases of the COVID‐19 pandemic. Prior to the pandemic, telehealth was a familiar term in New Zealand as it had been embraced in enabling access to specialist advice for clients who were separated from the providers due to geographical barriers [4, 6]. Mathematical modelling by Marani et al. suggests that the risk of another pandemic occurring in the next few decades or over a person’s lifetime is between 17% and 44% [21]. This risk is potentially increasing, and there remain opportunities for learning about the most efficient and effective way to utilise telehealth [22]. The international COVID‐19 pandemic increased the use of telehealth processes significantly throughout the world, and there has been an exponential increase in telehealth literature [23–25]. The findings from the current study further reinforce many of the conclusions of this published literature. This study also highlighted some unintended consequences, emphasising the importance of considering the context of the patient/client groups as well as professional/specialist needs regarding the consultation experience [26].

The aim of a face‐to‐face consultation with clients, patients and whānau (extended family) is to develop an empathic professional relationship while having a conversation about their health needs. These conversations typically include assessment, examination, exchange of information and the need for further investigations where relevant. The consultation occurs most commonly in the client’s own home or in a healthcare facility, with different professionals often having more experience in one or other of these contexts. The health professional should then use their clinical skills to effectively elucidate information of relevance to a client’s concerns before partnering with their client in the construction of a way forward or management plan.

It may be that the aim of a virtual synchronous telehealth consultation should be considered as subtly different or with different priorities; the healthcare professionals interviewed as part of this research worked in different contexts with reference to their professional environment, role and clients. Their relationship with telehealth during the pandemic is likely to have been influenced by their own personal, professional and contextual experiences, and there were some experiences described that resonated for many, while others were specific to a person, professional or context. As an example, the demographic of the clinicians may have influenced accessibility to telehealth, with younger clinicians who are more familiar with technology being more comfortable using certain modalities. The practice of healthcare staff was also heavily influenced by the politics within their organisation or profession, for example, staff employed by the District Health Board/Te Whatu Ora appeared more constrained by the protocols and what they could or could not do to facilitate patient care while MWs often felt enabled to act more individually with the women’s needs at the centre but within a framework of safe practice.

The provision of equitable care is a core aim of New Zealand’s current national health employer Health New Zealand (Te Whatu Ora) [27]. Factors influencing equity are many and complex and may be amplified by an increasing reliance on technology, particularly in those with minimal digital literacy [28, 29]. In this study, access to telehealth consultations was influenced by several factors (*Theme 1*); the geographical availability of infrastructure needed for wired or wireless connectivity as well as the need for the phone or devices to be shared between family members. There was also inequity of access to telehealth processes with a subtheme regarding cultural barriers that were present in both indigenous and immigrant populations, and while truly equitable healthcare for all may be elusive, equitable outcomes for all populations must be a priority, and telehealth may have a role [30]. Access within rest or nursing homes was also of concern, and it must be remembered that this was in the context of reduced visiting, which limited the assistance that families/whānau were able to give each other.

There was a heightened emotional awareness during the pandemic that was well documented. This was speculated to be a response to the perceived potential physical danger as well as to the actual psychosocial environment that was created [31, 32]. A driving force behind the use of personal protective equipment was the physical safety of healthcare staff, but there are other types of safety; some of the healthcare workers were fearful of not being able to do their role to the standard that they expected of themselves; they were concerned that clients/patients were not always engaging with services as they should have. The boundaries between work and home life became increasingly blurred (*Theme 5*) for many as they aimed at continuing to provide an adequate service under unfamiliar conditions. It was noted that the privacy of telehealth is not as guaranteed as an in‐person consultation, and there was concern about clients potentially not being in a safe environment where they could express themselves freely (*Theme 3*). It is an unfortunate fact that family violence is a real challenge for many people, and there is some evidence that this increased during the pandemic, particularly under conditions of lockdown [33] [34] [35] [36].

For some practitioners, experience in using telehealth, combined with heightened anxiety and pressure to learn during the COVID‐19 pandemic, was challenging, especially for those whose workload continued. The development of an effective infrastructure (*Theme* 6) has been well researched [37] and has been referred to in a recent publication as ‘creating conditions for practitioner implementation’ [38]. It was clear that guidelines written with the best of intentions by employers and professional organisations often contradicted each other and were not found to be written in a way that was *enabling* in order to promote the rapid development of processes and skills [39]. Some healthcare practitioners preferred the accessibility of information provided by colleagues on social media platforms (‘Twitter’/‘Facebook’) and often used their own time and resources to develop effective telehealth processes, reflecting the concept that work often happens in a very different way (‘work as done’) to that prescribed by authorities or managers (‘work as imagined’). There were significant innovations by all staff (*Theme 4*).

Humans require a personal connection, based on interactions over time that are informed by a variety of senses, not just verbal communication [40]. Many of those interviewed also talked about the challenges in establishing an effective professional relationship with clients that they had not previously met (*Theme 2*). Communication over telehealth limits the availability of some types of information to develop this relationship, and this was noted to be particularly hard when there was a language barrier and a need for an interpreter. There were fewer concerns about continuing a professional relationship that had already been established. Comments suggested that some staff were now more comfortable with the use of a virtual consultation to provide feedback about reassuring results (both screening and diagnostic), rather than arranging an in‐person review. It is also interesting to note that in this study, the telephone was the most common choice for the client regarding modality of contact, which further restricts the availability of visual communication such as facial expression. Conversely, there is some evidence that the use of video, particularly in education and conference calls, contributes to exhaustion (or Zoom Fatigue) because of effects such as excessive videoconferencing, ‘mirror anxiety’ (from seeing their own self‐image) and ‘hyper gaze’ from feeling watched [41]. These factors influenced the structure of the telehealth consultation and the style of verbal communication that occurred within it (*Theme 3*). At times, practitioners had to be quite directive in describing the format of the consultation and the time limitations (whereas these would have previously been inferred in the more familiar face‐to‐face communication contexts) or that a face‐to‐face consultation would be necessary to perform important aspects of the examination. At times, clients/patients perhaps did not make contact early enough, which was likely to have occurred because of factors such as not wanting to be exposed to COVID‐19 and being aware of the workload of each healthcare practitioner. Further potential challenges for the clients included an assumption that because the country was in lockdown, they were physically available and could be called at any time, whereas many of them had multiple demands on their time; these could have included working at home, partners working from home and children attending virtual school lessons (*Theme 4*).

A predictable benefit of the use of telehealth was that clients did not have the inconvenience of driving to a healthcare facility, parking and waiting to be seen. They were predominantly in their own homes, and although they were potentially busy (as above), they were also able to talk on the telephone while observing family members in their care. There were also several potential benefits to the use of telehealth that were not anticipated; these included the evaluation of children under developmental surveillance; these children were able to be observed within their own homes with their own possessions, and in some cases, the children were less inhibited using this modality, enabling the practitioner to evaluate their developmental progress (*Theme 3*). Their parents were also able to then arrange to talk privately with the healthcare practitioner. In some other paediatric consultations where parents had separated, both parents felt more able to attend using telehealth than they would have if they had been present in the same room. This concept of a telehealth conference also at times made discussions between professionals easier to organise because the meeting was held in a ‘virtual’ space rather than at the location of one member of the team. However, there was also unfortunately some evidence that barriers between professionals became exacerbated during lockdown; the different professional organisations and employers did not always work together to enable patients/clients under these unusual conditions, which created boundaries (*Theme 5*) at various professional and departmental interfaces.

## 5. Limitations

This work occurred in a specific timeframe characterised by initial lockdown secondary to the COVID‐19 pandemic, at a time when rates of COVID‐19 were low in the New Zealand community. Using a pragmatic approach to sampling, not all healthcare professional groups were represented by those interviewed. It is hoped that by including a range of professions, we have covered a broad range of experiences. The results of this study may not be generalisable outside the New Zealand healthcare setting, although they may generalise to other similar health systems such as wider Australasia. The COVID‐19 pandemic was also unique in that the nationwide lockdown provided specific healthcare challenges, which may differ in other contexts. However, the themes identified raise interesting perspectives that may then be further explored in other settings.

## 6. Conclusion

The themes identified as part of the telehealth experiences explored within this research provide insight into the further preparation required in the provision of synchronous telehealth for a future pandemic. A feature of major healthcare crises is that they can reveal the precipitating fault lines that have preceded and contributed to the event. This can present unique opportunities to investigate and develop improved preparedness strategies and a better plan going forward. It is hoped that the results from this qualitative research have relevance in other interprofessional healthcare situations, as our research team sought to continually improve their interprofessional understanding as practitioners and researchers. The six themes have further validated the importance of considering the complexity of context regarding the client, practitioner, their professional relationship and the needs of the consultation and providing the right conditions for effective practitioner implementation.

As New Zealand embraces a new national healthcare system, there must be mature recognition of how to effectively blend the virtual consultation with the face‐to‐face consultation in ways that nurture our workforce [42] and maintain or enhance equity but also improve efficiency [43]. Professional organisations and employers need to recognise how to develop their leadership to support staff in their provision of care, in spite of workload and changes to routine systems of work. This infrastructure of support and interprofessional preparedness is essential as we continue to use telehealth as a tool for patient care and as we prepare for future challenges. By identifying these core themes, we provide a foundation for clinical resilience that ensures telehealth systems are not merely reactive but are robustly prepared for the inevitable disruptions of future healthcare crises.

## Author Contributions

All authors contributed to the research and to the writing of this paper.

## Funding

The study was funded by the University of Canterbury (10.13039/100008414). Open access publishing facilitated by University of Otago, as part of the Wiley ‐ University of Otago agreement via the Council of Australasian University Librarians.

## Ethics Statement

This research was educational research conducted with healthcare practitioners’ ethical approval (HEC 2020/52), which was received in September 2020 from the University of Canterbury Human Ethics Committee, and updates to this to allow interviewees to be from outside of Canterbury were also subsequently approved. An information sheet and consent form were provided for those being interviewed, who were made aware that the only personal information collected was their profession and gender.

## Conflicts of Interest

The authors declare no conflicts of interest.

## Data Availability

The datasets generated and/or analysed during the current study are not publicly available due to the small sample size and potential for identification of individuals but are available from the corresponding author on reasonable request.

## Appendix 1: COREQ Checklist

**Table A1:** Consolidated criteria for reporting qualitative studies (COREQ): 32‐item checklist.

Item no.	Guide questions/description	Reported on page #
Domain 1: Research team and reflexivity		
Personal characteristics		
1. Interviewer/facilitator	Which author/s conducted the interview or focus group?	p. 7
2. Credentials	What were the researcher’s credentials? e.g., PhD and MD	p. 9
3. Occupation	What was their occupation at the time of the study?	p. 9
4. Gender	Was the researcher male or female?	p. 9
5. Experience and training	What experience or training did the researcher have?	p. 9
Relationship with participants		
6. Relationship established	Was a relationship established prior to study commencement?	p. 7
7. Participant knowledge of the interviewer	What did the participants know about the researcher? e.g., personal goals and reasons for doing the research?	p. 7
8. Interviewer characteristics	What characteristics were reported about the interviewer/facilitator? e.g., bias, assumptions, reasons and interests in the research topic	p. 8
Domain 2: Study design		
Theoretical framework		
9. Methodological orientation and theory	What methodological orientation was stated to underpin the study? e.g., grounded theory, discourse analysis, ethnography, phenomenology and content analysis	p. 8
Participant selection		
10. Sampling	How were participants selected? e.g., purposive, convenience, consecutive and snowball	p. 7
11. Method of approach	How were participants approached? e.g., face‐to‐face, telephone, mail and email	p. 7
12. Sample size	How many participants were in the study?	p. 9
13. Nonparticipation setting	How many people refused to participate or dropped out? Reasons?	n/a
14. Setting of data collection	Where was the data collected? e.g., home, clinic and workplace	p. 7
15. Presence of nonparticipants	Was anyone else present besides the participants and researchers?	n/a
16. Description of sample	What are the important characteristics of the sample? e.g., demographic data and date	p. 9
Data collection		
17. Interview guide	Were questions, prompts and guides provided by the authors? Was it pilot tested?	p. 7
18. Repeat interviews	Were repeat interviews carried out? If yes, how many?	n/a
19. Audio/visual recording	Did the research use audio or visual recording to collect the data?	p. 7
20. Field notes	Were field notes made during and/or after the interview or focus group?	n/a
21. Duration	What was the duration of the interviews or focus group?	p. 10
22. Data saturation	Was data saturation discussed?	p. 7
23. Transcripts returned	Were transcripts returned to participants for comment and/or correction?	p. 8
Domain 3: Analysis and findings		
Data analysis		
24. Number of data coders	How many data coders coded the data?	p. 8
25. Description of the coding tree	Did the authors provide a description of the coding tree?	n/a
26. Derivation of themes	Were themes identified in advance or derived from the data?	p. 8
27. Software	What software, if applicable, was used to manage the data?	n/a
28. Participant checking	Did participants provide feedback on the findings?	n/a
Reporting		
29. Quotations presented	Were participant quotations presented to illustrate the themes/findings? Was each quotation identified? e.g., participant number	pp. 10–15
30. Data and findings consistent	Was there consistency between the data presented and the findings?	pp. 15–20
31. Clarity of major themes	Were major themes clearly presented in the findings?	pp. 10–20
32. Clarity of minor themes	Is there a description of diverse cases or a discussion of minor themes?	pp. 10–20
